# Orthopedic Surgery in Ambulatory Surgery Centers During the COVID-19 Pandemic: Low Incidence of Infection Among Patients, Surgeons, and Staff

**DOI:** 10.7759/cureus.24247

**Published:** 2022-04-18

**Authors:** Paul R Diekmann, Owen R O’Neill, Edward R Floyd, Laura C Meinke, Justina Lehman-Lane, Rachel M Uzlik, Rebecca Stone McGaver

**Affiliations:** 1 Orthopedic Surgery, Twin Cities Orthopedics, Golden Valley, USA; 2 Orthopedic Surgery, Revo Health, Golden Valley, USA; 3 Orthopedic Surgery Research, Twin Cities Orthopedics, Golden Valley, USA

**Keywords:** epidemic, universal testing, symptom tracking, screening, outpatient, asc, covid-19

## Abstract

Background and objective

The coronavirus disease 2019 (COVID-19) pandemic has presented tremendous challenges to the healthcare systems worldwide. Consequently, ambulatory surgery centers (ASCs) have been forced to find new and innovative ways to function safely and maintain operations. We conducted a study at a large United States (US) private orthopedic surgery practice, where a universal screening policy and testing protocol for COVID-19 was implemented for patients and ASC personnel including surgeons, in order to examine the incidence of COVID-19 in patients scheduled for orthopedic surgery in ASC settings as well as the incidence among the surgeons and ASC personnel.

Methods

The universal screening protocol was implemented in the ASCs of the facility during the early stage of the pandemic for an eight-month period from April 28, 2020, to December 31, 2020. All ASC personnel including surgeons had their symptoms tracked daily and were rapid-tested every two weeks. All patients were screened and tested before they entered the ASC.

Results

A total of 70 out of 12,115 patients and 41 out of 642 ASC personnel tested positive for COVID-19, resulting in infection rates of 0.6% and 6.4%, respectively. Individual symptoms, age, the American Society of Anesthesiologists (ASA) scores, and comorbidities were documented, and no single factor was found to be common among positive (+) tests.

Conclusions

The implementation of universal screening and symptom-reporting procedures was associated with a very low rate of infections among ASC patients, staff, and surgeons, and it offers a reproducible framework for other facilities to continue to provide orthopedic outpatient operations in ASC settings during the ongoing iterations of the COVID-19 pandemic.

## Introduction

The outbreak of the coronavirus disease 2019 (COVID-19) pandemic, caused by severe acute respiratory syndrome coronavirus 2 (SARS-CoV-2), has challenged healthcare systems worldwide at every level, and authorities have been forced to implement new infection control procedures and epidemiological tracking. As of April 12, 2022, there have been over 80 million infections in the United States (US), and over 6.1 million deaths worldwide due to COVID-19 [1,2]. In an effort to conserve resources, including critically limited personal protective equipment (PPE), elective surgeries throughout the US were stratified based on their urgency during the peak of the pandemic [3,4]. During this time, the focus of orthopedic surgery departments worldwide shifted to infection control, treatment of urgent musculoskeletal injuries, and resource conservation [5-10].

As vaccination rates in the US increased (82.1% of adults have received at least one dose as of April 12, 2022), the rate of new infections decreased, and COVID-19-related restrictions were gradually lifted. Following this, orthopedic ambulatory surgery centers (ASCs) began to operate at full capacity again and mask mandates for patients began to be relaxed [2]. However, the pandemic continues to plague other parts of the world, and other forms of the disease are becoming predominant. Epidemiological data from war-torn regions from Ethiopia and Yemen to Syria remain scarce, and while global positivity rates decrease, some areas are experiencing resurgences fueled by COVID-19 variants of concern (VOCs) [11-13].

New variants of COVID-19 have spread throughout the world, viz, B.1.1.7 (alpha), B.1.351 (beta), B.1.617.2 (delta), and recently, the BA.1 (Omicron) and its subvariant BA.2 [14]. The Omicron variants have become the dominant variant in the US, and they appear to be more transmissible albeit less severe than the prior variants. Reviewing evidence of successful past efforts at containment control will be valuable to nations still undergoing COVID-19 outbreaks, and will serve as important records of the efforts to contain this pandemic, which they could rely on and learn from if future respiratory viral outbreaks or COVID-19 VOCs should generate worldwide concern [15,16].

Guidelines have emerged from different regions of the world as the virus rapidly spread. In Singapore, the previous pandemic experience enabled the public health sector to provide adequate quarantine facilities and an extant response network to combat the subsequent outbreaks, allowing orthopedic surgeons to focus on triage, patient and staff protection, and conservation of healthcare resources while continuing elective day-surgery cases throughout the pandemic [6]. Researchers from Bergamo, Italy have laid out a multilevel safety approach consisting of universal symptom screening among all staff and patients, routine self-questionnaires, temperature checks, mask-wearing, social distancing, reduction of visitors in waiting rooms, digital pre-recording of histories before physical interaction, and digital payments/scheduling to minimize document exchange [7].

Safely reopening orthopedic ASCs and resuming elective surgical procedures was seen as a critical early objective, as delaying many elective procedures carried a significant long-term impact on patients’ quality of life [3,17]. It is therefore important to examine the role of time-sensitive and elective surgery under pandemic conditions in orthopedic ASCs as guidelines for operational safety have become more refined. Additionally, ASCs account for a significant portion of the revenue of many academic departments and private orthopedic practices. Keeping outpatient ASCs open through ongoing iterations of the virus and future pandemic scenarios will be instrumental in mitigating elective-case cancellations and minimizing the associated financial losses that accompany lockdowns. Preliminary evidence has shown that the safe functioning of orthopedic ASCs with the use of universal testing, screening for COVID-19 symptoms, and clear and rigid guidelines for infection control procedures is a viable option [12,18-21]. The success of outpatient centers under these circumstances may provide a blueprint for utilizing the ASC setting as a safe alternative to elective surgery in hospital settings under pandemic or resource-limited conditions, where hospital resources have to cater to higher levels of urgency.

Early on in the pandemic, a ban on elective surgeries temporarily went into effect in the US State of Minnesota on March 16, 2020 [12]. The leadership of a large private orthopedic practice in the US quickly developed and subsequently implemented a universal testing protocol by utilizing available guidelines for patients undergoing time-sensitive elective orthopedic surgery in their ASCs [12,22-24]. Simultaneously, the practice developed and implemented an additional universal testing protocol for the ASC surgeons/personnel. Resumption of time-sensitive elective surgery in the ASCs was dependent upon the ability to implement screening measures and protocols addressing the long incubation time and asymptomatic spread of the disease [16,25,26]. Both protocols continued to be in place even after the statewide ban on elective surgery was lifted on May 5, 2020, with protocols maintained through May 2021 [12]. This study reports the incidence of COVID-19 in patients scheduled for orthopedic surgery in ASC settings as well as the incidence among the surgeons and ASC personnel. This analysis of COVID-19 incidence in ASCs before the widespread availability of vaccines should prove useful for studying future pandemic or VOC response methodology and overall preparedness.

## Materials and methods

This level III study was approved by the IntegReview Institutional Review Board. Prospective data collection was conducted between April 28-December 31, 2020, and was retrospectively reviewed to identify all consecutive patients who underwent time-sensitive elective surgery by Twin Cities Orthopedics (TCO) surgeons within one of the four ASCs managed by Revo Health. A universal screening policy and testing protocol for COVID-19 implemented for the surgeons and ASC personnel were also reviewed to assess the incidence of infection and prevalence of symptoms. ASC personnel was defined as surgeons, physicians’ assistants, anesthesia teams, and staff (all ASC employees, including non-clinical support staff).

Each ASC adopted and employed identical policies and COVID-19 screening processes/protocols to screen all individuals entering the ASC, in accordance with the US Federal Government, Centers for Disease Control and Prevention (CDC), and the Minnesota Department of Health guidelines instituted during the initial period of the global transmission of COVID-19. Policies limited point of entry, and prevented or mitigated exposures in each ASC. All patients, ASC personnel, and others entered the facility at the screening point and participated in the COVID-19 screening process.

ASC testing

ASC personnel completed an online daily screening assessment (Proventus Health, Inc., Golden Valley, MN) and bi-weekly COVID-19 reverse transcription-polymerase chain reaction (RT-PCR) test (OralDNA Labs, Eden Prairie, MN). Additionally, all personnel self-monitored daily for fever, cough, and shortness of breath, and avoided travel to high-risk areas and close contact with people who were sick. If individuals had a fever (temperature of 100.4 ℉ or higher), cough, or shortness of breath, they refrained from entering the ASC and underwent testing at one of the organization’s contactless testing centers. Screening and mandatory testing for vaccinated staff ended at the end of January 2021.

Patient testing

All patients who had time-sensitive surgeries underwent a COVID-19 test prior to surgery with no post-discharge test. An RT-PCR test was done no more than 96 hours prior to their scheduled procedure with the patients practicing self-quarantine between the test time and arrival at ASC. Testing was performed at one of the seven testing centers within the group’s offices. If a patient missed their collection time, the surgery was rescheduled until the COVID-19 test was performed. Test results were faxed to the ASC and patients would receive a call if they tested positive. Patients who tested positive were all contacted to reschedule at a later date. Patient testing centers utilized a combination of drive-up or walk-in; employees performing the test wore the full PPE gear (N-95 mask, eye-protection/face-shield, clean scrubs, disposable gown, scrub cap, and disposable gloves).

Upon arrival for surgery, patients were screened for symptoms including fever (100.4 ℉ or higher), new-onset cough, and/or shortness of breath before access beyond the facility lobby was granted. A screener (ASC staff) was stationed in the lobby with surgical masks, gloves, and hand sanitizer; they documented the procedure manually using the COVID-19 Screening Log. Part of the safety profile was the restriction of traffic. Visitors were not allowed to enter with the patients; however, minor patients (under 18 years of age) could be accompanied by one family member. Designated drivers for patients could not enter the building. Drivers were called by the post-anesthesia care staff to meet patients outside the building when they were ready for discharge. Mandatory COVID-19 patient testing continued until June 1, 2021, when vaccinated patients were no longer required to receive screening before surgery.

Data

Data collection specific to ASCs included the number of surgeons and ASC personnel tested, number of positive tests, location, and individual symptomatology based on daily Proventus tracking information. Patient data included age, gender, smoking status, comorbidities, the American Society of Anesthesiologists (ASA) scores, and the type and current procedural terminology (CPT) code of surgery.

## Results

Patients

**Figure 1:**
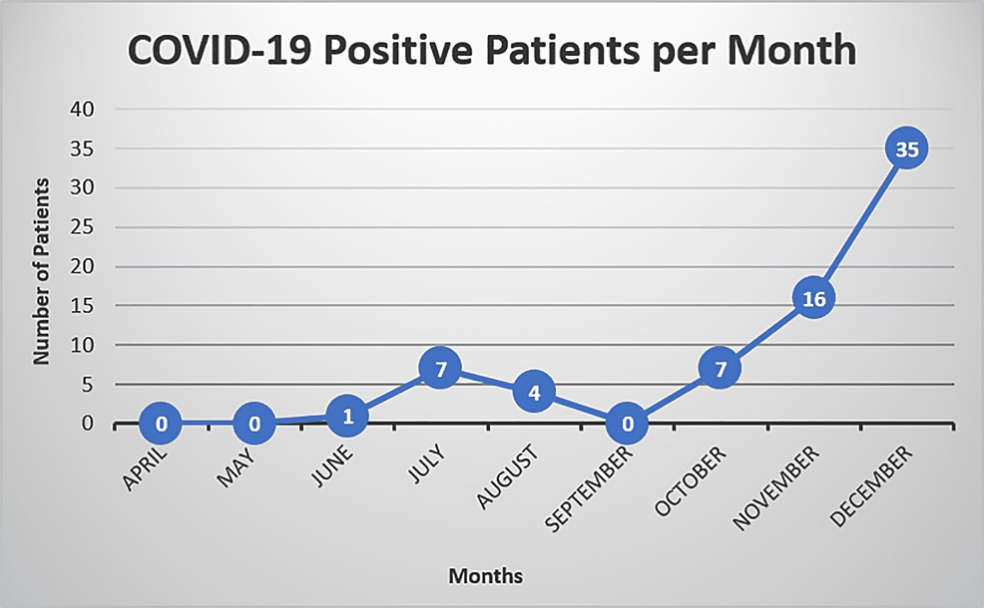
COVID-19-positive patients per month Representation of patients who tested positive for COVID-19 during the study period of April-December 2020 COVID-19: coronavirus disease 2019

**Figure 2:**
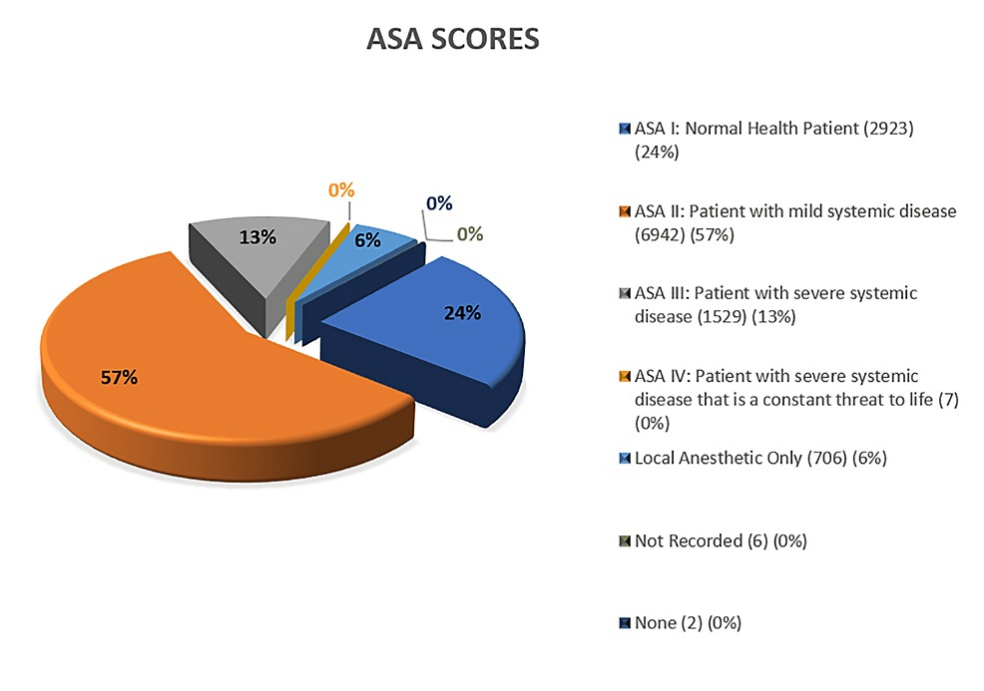
American Society of Anesthesiologists (ASA) scores The American Society of Anesthesiologists (ASA) scoring for all patients during the study period; 57% (6,942 patients) were ASA class II (mild systemic disease), followed by normal healthy patients (ASA class I, 24%). There were seven patients with severe systemic disease causing constant threat to life (class IV), and no higher-grade or emergency patients. Twenty-five COVID-19-positive patients were ASA class 1, otherwise healthy patients; 27 were ASA class 2; 10 were ASA class 3; three were local anesthesia-only, and the status of five patients could not be determined

During the period examined, 12,115 patients underwent 13,304 surgical procedures on the basis of time-sensitive orthopedic complaints. Females represented 53.6% (n=6,491) of the patient population. The average patient age at the time of surgery was 52 years (SD=17.4). There were 70 unique patients who tested positive for COVID-19, representing an incidence rate of 0.6% (Figure 1). The ASA scoring is illustrated in Figure 2.

**Figure 3:**
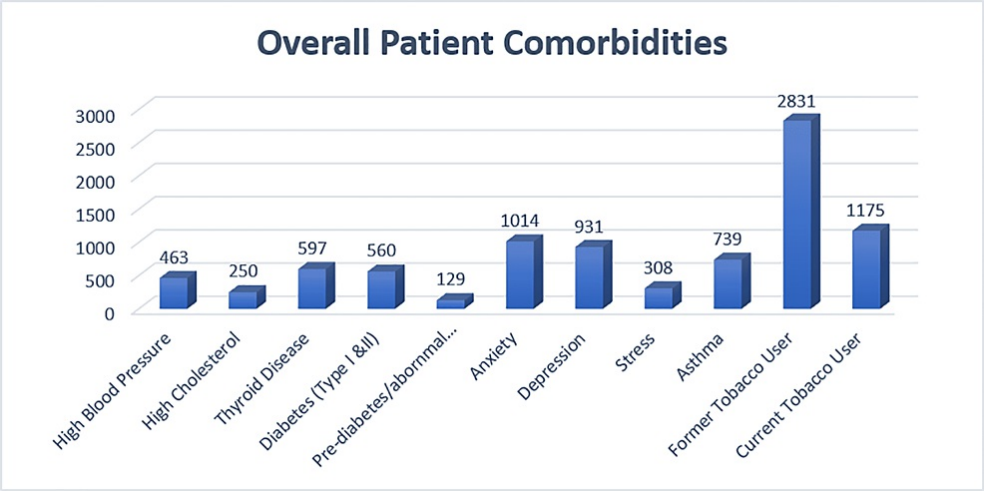
Overall patient comorbidities Comorbidities among the entire patient population. Anxiety and depression represented the most commonly prevalent comorbidities in our patient population

Most of the patients (57%) were ASA class II, followed by ASA class I (24%). Patient comorbidities are depicted in Figure 3.

A sizeable proportion of the patients were current or former tobacco users (33.1%), and 6.1% had a history of asthma. Of the 739 patients who had asthma, four were COVID-19-positive.

**Figure 4:**
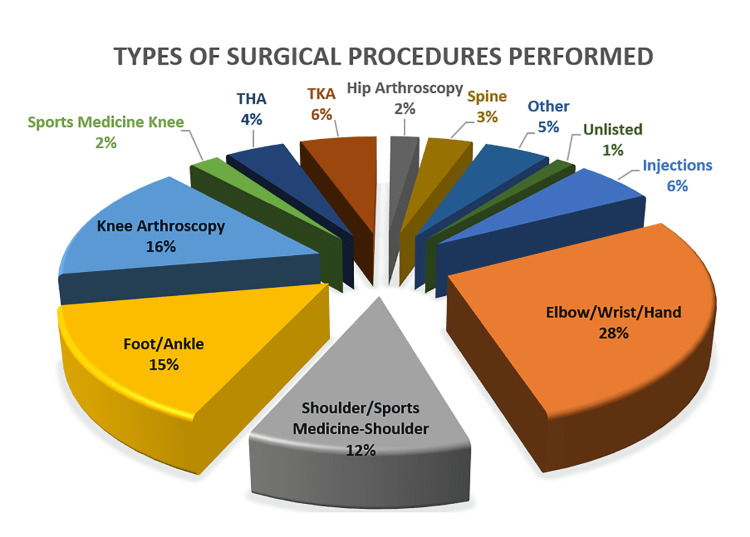
Types of surgical procedures performed Types of surgery performed during the study period. Elbow, wrist, and hand procedures made up the highest proportion of surgeries carried out during the study period (28% overall). Total hip and knee arthroplasties made up a combined 10% of total surgeries THA: total hip arthroplasty; TKA: total knee arthroplasty

Figure 4 demonstrates the procedures performed upon the categorization of the surgical type and CPT code; elbow, wrist, and hand procedures were the most common ones (28%), followed by knee arthroscopy (16%) and foot and ankle surgeries (15%).

COVID-19-positive patients

All patients who tested positive were rescheduled at the ASCs at an average of 3.4 weeks after their original date of surgery, except three patients (two had surgery at the hospital and one rescheduled at an outside facility). The average age of the 70 patients who tested positive was 46 years (range: 15-77 years). Forty were male. There were six current tobacco users (8.6%) and 12 former tobacco users (17.1%). The most common comorbidities were hypertension (7.1%) and asthma (5.7%), followed by hyperlipidemia, anxiety, and depression (4.3% each).

Twenty-five COVID-positive patients were ASA class 1, otherwise healthy patients; 27 were ASA class 2; 10 were ASA class 3; three were local anesthesia-only, and the status of five patients could not be determined. Patients who tested positive were generally asymptomatic prior to testing and were detected through the universal screen.

ASC personnel

For the 642 ASC personnel, 8,594 COVID-19 tests were performed, one every two weeks. Each ASC personnel was serially tested an average of 13.1 times (Table 1).

**Table 1:** Serial testing count for ASC personnel ASC: ambulatory surgery center

Position	Average number of tests per person	Minimum number of tests per person	Maximum number of tests per person
ASC staff	13.6	1	23
Surgeons	14.0	1	21
Physician assistants	13.9	1	21
Anesthesiologists	10.9	1	21
Average for ASC personnel	13.1		

There were 48 positive tests as delineated in Table 2, with 41 unique individuals testing positive. The incidence of COVID-19 among ASC personnel was 6.4% (41/642), including seven positive tests among surgeons.

**Table 2:** COVID-19 tests and results among ASC surgeons, staff, and associates ASC: ambulatory surgery center; COVID-19: coronavirus disease 2019

Position	Number of tests	Number of individuals	Number of positive tests	Number of individuals
ASC staff	4,701	345	24	20
Surgeons	1,416	100	7	7
Physician assistants	1,360	97	8	7
Anesthesiologists	1,117	100	9	7
Total ASC personnel	8,594	642	48	41

**Figure 5:**
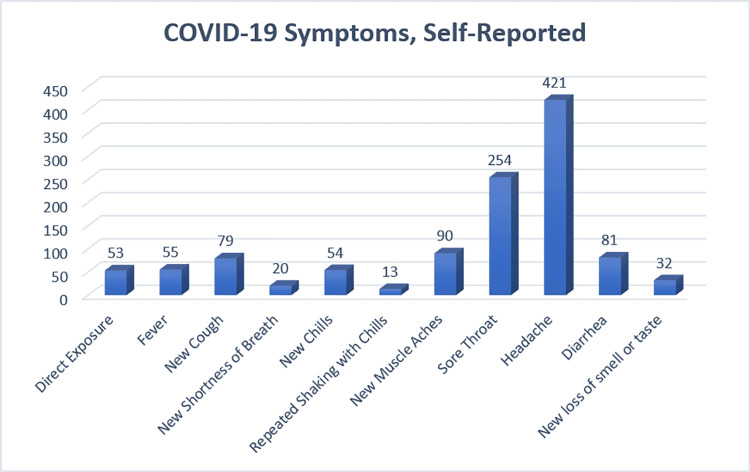
COVID-19 symptoms, self-reported Symptoms of COVID-19 reported among ASC personnel and surgeons pooled from the four ASCs. These data were gathered from a screening form sent electronically to each employee every day. The most common symptoms associated with COVID-19 reported among employees were headache (421) and sore throat (254) ASC: ambulatory surgery center; COVID-19: coronavirus disease 2019

The screening data for ASC personnel and surgeons are detailed in Figure 5.

The most common symptoms reported were “headache” and “sore throat”. Fifty-three surgeons and ASC personnel reported positive exposures to COVID-19-infected individuals. Sixteen of those 53 also reported some combination of individual symptoms. Of these, “headache” (12/16) was again the most common, followed by “new muscle aches” (9/16).

## Discussion

Based on our findings, the infection control procedures implemented at the ASCs were associated with a low incidence of COVID-19 in patients and personnel. Universal testing revealed that 70 patients (0.6%) scheduled for surgery were positive for COVID-19 before entering the ASC.

While the number of positive patients and employees may represent an insufficient data set from which to draw broad conclusions about the general characteristics prevalent among COVID-19 patients, we feel that the incidence demonstrates the effectiveness of the universal screening program in its ability to detect infections among ASC personnel and patients. A 2021 study from Italy has described a similar RT-PCR universal testing protocol for screening patients before they underwent hospital-based inpatient surgical procedures [27]. Among 622 patients scheduled for orthopedic surgery, COVID-19 infections were preoperatively detected in eight asymptomatic patients, representing an incidence rate of 1.3%. There were no positive tests among surgeons or healthcare workers [27]. Another 2021 study analyzed the incidence of COVID-19 cases among patients and hospital staff who were in contact with them in one of the largest referral orthopedic hospitals in Iran [28]. Patients were screened for COVID-19 before surgery and tested, along with the hospital staff, four weeks after discharge. The incidence of COVID-19 was 1.9% in patients and 5.5% in hospital staff [28]. To our knowledge, this report is the first investigation of COVID-19 incidence among the patients, staff, and surgeons in outpatient orthopedic ASCs. It is also the first report of a surveillance symptom-tracking program, serial testing, and a universal screening protocol for personnel in orthopedic ASC settings; this could provide valuable insights into being prepared for future pandemics.

Among ASC personnel who endorsed symptoms and/or tested positive, the most common symptom was headache. The sampling was small and therefore may not be expected to correspond to trends in the broader population. For instance, a sampling from the CDC in July of 2020 showed quite a different symptom prevalence, with “headache” reported in just 59% of the confirmed cases, while “cough” was the most prevalent symptom, endorsed by 84% of the patients [29]. We believe that this variability among our patients demonstrates the importance of testing every patient before they undergo procedures, as well as testing and screening employees for any symptoms and any contacts as there is no such thing as a “standard” COVID-19 patient.

As COVID-19 restrictions are being gradually eased in the US, many regions in the world are still suffering from resurgent COVID-19 VOCs. A slower rate of vaccination along with a lack of reporting and testing availability in the developing world and in countries with ongoing geopolitical instability means that continuing mutations of the virus will likely pose a risk for resurgence for some time to come. Moreover, pandemic response plans were heterogeneous in their methodology and effectiveness across the world at the outbreak of the current pandemic. In 2020, elective surgery at ASCs was paused around the world as health systems had to focus on triage and conservation of resources. Results of this study suggest that orthopedic ASCs, by using universal testing for patients, continuous testing and monitoring of healthcare personnel, and infection control procedures such as those outlined here, may provide a safe alternative for ongoing elective surgery in the outpatient setting, even as elective cases may be paused in hospital settings.

The gathering of these data was initially performed during the early period after the COVID-19 shutdown and continued through the end of 2020. The trends in incidence of COVID-19 have changed dramatically nationwide since these data were collected, with the incidence both decreasing and increasing over time since the study period in the region of the authors’ practice. The patient safety protocols remained in place from the initiation of the study period through May 2021, when patients were allowed to begin visiting the ASCs with optional mask-wearing. The masking mandate ended as the vaccination rates among the population increased and the incidence of COVID-19 in the state of Minnesota decreased [30]. In regions where COVID-19 presents an ongoing hazard and as a preparation for future respiratory viral outbreaks, these procedures may provide a blueprint for continuing time-sensitive elective surgeries in an orthopedic outpatient setting [31,32]. Indeed, in some areas, public authorities are calling again for a reduction in elective surgery procedures [32]. As new variants of COVID-19 continue to evolve, the ongoing pandemic response procedures may be warranted in the outpatient setting to maintain avenues available for safe and timely elective orthopedic surgeries.

Limitations of this study mainly pertain to the age and ASA scores of the patients who had surgery at ASCs, as well as patient self-reported data. The average age of patients (52 years) and predominant ASA scores of I and II are reflective of the relative health status of ASC patients versus the general population. It should be noted that even among younger, healthy patients, return to baseline and resolution of symptoms are often prolonged, according to the interviews conducted by the CDC [33]. This is reflected in our COVID-19-positive patients, whose ages ranged from 15 to 77 years. Another limitation was the lack of availability of patient self-reported symptom data. Patients self-reported comorbidities on their initial clinic intake forms, and employees self-reported their daily symptoms. Self-reporting may potentially provide another source of error. The COVID-19-positive subjects in this study represented the first two waves of the pandemic only, even though, as previously noted, the goal of this study was to provide a generalizable template for adapting to future pandemics.

## Conclusions

The ongoing COVID-19 pandemic has caused a tremendous burden on healthcare systems around the world, and hence ASCs have been forced to adapt to it by finding new ways to function safely and maintain operations. We conducted this study at a large US private orthopedic surgery practice where a universal screening policy and testing protocol for COVID-19 was implemented for patients and ASC personnel including surgeons. The execution of these universal screening and symptom-reporting procedures was associated with a very low rate of infections among ASC patients, staff, and surgeons. Moreover, this system offers a reproducible framework for providing orthopedic outpatient surgical procedures in an ASC setting during the ongoing iterations of the COVID-19 pandemic.
